# Characterization of multi-targeted insulin-mimetic antidiabetic peptides using *in silico* approaches

**DOI:** 10.1371/journal.pone.0330341

**Published:** 2025-08-19

**Authors:** Anas Bilal, Ghulam Mustafa

**Affiliations:** Department of Biochemistry, Government College University Faisalabad, Faisalabad, Pakistan; Institute of Medical Sciences, Banaras Hindu University, INDIA

## Abstract

Diabetes is a metabolic disease that can affect people at any age. Despite being one of the leading causes of death, the treatment of diabetes is extremely difficult. For the treatment of diabetes mellitus various synthetic oral hypoglycemic drugs and insulin is available. However, insulin cannot be taken orally and the synthetic agents used can have harmful side effects. Bioactive peptides are particular protein fragments that have a beneficial impact on human health and physiological processes. These peptides can be applied as antidiabetic agents in the treatment of diabetes. The aim of the current study was to develop hypoglycemic peptides (HGPs) from plant sources and to investigate their binding interactions with selected diabetic protein receptors using peptide-protein docking, in order to identify potential peptide candidates for the treatment of diabetes mellitus. The binding patterns of top seven hypoglycemic peptides with ten selected receptor proteins were explored using peptide-protein docking. The peptides P2 and P6 showed best haddock scores and binding pattern against target receptors. The P2 showed strong interactions with maltase-glucoamylase (binding energy of −108.7 + /- 9.3 kcal/mol and with six hydrogen bonds), glucose transporter 1 (GLUT1) (binding energy of −85.3 + /- 1.4 kcal/mol and with three hydrogen bonds). Peptide 6 exhibited highest score against insulin-like growth factor 1 receptor (binding energy of −107.5 + /- 5.4 kcal/mol and with seven hydrogen bonds) and pancreatic alpha-amylase (binding energy of −121.3 + /- 3.1 kcal/mol and with seven hydrogen bonds), while against Jun N-terminal kinase1 receptor peptide, the peptides P5 and P6 showed same haddock energy of −92.1 + /- 4.1 kcal/mol having different number of hydrogen bond interactions in both complexes. The molecular dynamics simulation revealed that P6 and P2 were firmly bound to insulin-like growth factor 1 receptor and maltase-glucoamylase, respectively for the simulation time of 100 ns. The findings of this computational study support the data showing that these hypoglycemic peptides are effective against selected diabetic proteins. The insulin-mimetic antidiabetic peptides would be integrated into active packaging systems for the development of multi-functional materials to regulate glucose absorption. However, further assessment and validation of the particular peptides as potential therapeutic candidates for diabetes mellitus are required.

## Introduction

Diabetes mellitus (DM) is recognized as a prevalent multifactorial disease that is approaching epidemic proportions. It has been affecting every age group without any discrimination [1]. Despite being one of the leading causes of death, treatment of diabetes is extremely difficult [2]. Diabetes is characterized by signs including glucose intolerance, prolonged hyperglycemia and disruption in regulatory systems that store and utilize metabolic energy such as carbohydrate, lipid, and protein catabolism and anabolism in a diabetic individual due to impaired insulin receptor functioning or lack of insulin production [3].

Type 1 diabetes is typically diagnosed in people under the age of 18, but type 2 diabetes is more common in adults and the elderly. Roughly 10% of all diabetes diagnoses are type 1 with the remaining 90% being type 2 [4]. Diabetes is becoming increasingly common and the problem is projected to worsen as a result of current lifestyle choices. Type 2 diabetes affected 422 million people in 2017 and is estimated to reach 642 million by 2040. According to the data, the number of diabetic persons will increase nearly 50% over the next two decades. Diabetes is a problem because one person dies from it every six seconds [5].

Although insulin and many artificially synthesized oral hypoglycemic medications are available to treat diabetes, but insulin cannot be taken orally and the synthetic medicines now in use can cause substantial toxicity and side effects [6]. In many regions of the world, medicinal plants are being utilized to treat diabetes and there is a hidden treasure of potentially beneficial natural products that can control diabetes [7]. Different conventional antidiabetic plant remedies are thought to have less adverse effects and less harmful than synthetic drug therapies [8]. More than 800 plants are said to have antidiabetic effects and to treat hypoglycemia an ethnopharmacological review identified over 1200 herbs used in traditional medicine [9].

Form plant and animal sources, several bioactive peptides have been reported. Most bioactive peptides are sourced from animal products such as meat, fish, eggs, and milk. Plant bioactive peptides are still being investigated and a small number of peptides have been discovered from wheat and soy [10]. The utilization of plants or herbal sources is gaining popularity for the development and discovery of new antidiabetic drugs that can control diabetes having least side effects than conventional drugs [11]. Bioactive peptides are particular fragments of protein(s) that improve physiological functioning as well as human health [12]. The peptides have different characteristics including high target bioactivity, less toxicity, reduced incidence of tissue aggregation with in the body, high structural diversity, and smaller size (e.g., antibodies) [13]. These characteristics make peptides suitable as therapeutic drugs with various properties such as antidiabetic, antibacterial, antihypertensive, antithrombotic, and antioxidant properties. Bioactive peptides produced from plants can manage glucose levels in blood, cause reduction in insulin resistance, and inhibit diabetes [14].

The traditional and single-target drugs are often failed in addressing the interconnected mechanisms of insulin resistance, β-cell dysfunction, and hyperglycemia, which lead to side effects and restricted efficacy [15]. The in silico methods to discover antidiabetic peptides would overcome limitations of conventional drug discovery (e.g., high costs and long development timeline) by prioritizing antidiabetic agents for experimental validation. The integrative computational approaches accelerate the process of drug discovery of multi-target antidiabetic peptides with enhanced efficacy and reduced off-target effects, which could be a promising alternative in traditional therapies [16]. The purpose of the current study was therefore to delve into and discover hypoglycemic peptides found in different medicinal plant proteins. First, bioinformatics methods were used to identify efficient peptides, which were then analyzed for physical qualities and binding to specific target proteins by molecular docking approach.

## Materials and methods

### Protein sequences

Based on the literature information, the protein sequences of plant hypoglycemic proteins including insulin mimetic protein (also known as Cq-IMP) (UniProt accession number: C0HLQ3), AdMc1 protein (UniProt accession number: A0A0M5WZ27), polypeptide-P (UniProt accession number: ADO14327), prolamin binding factor (UniProt accession number: OP763567), MC6 (UniProt accession number: AAX06814), charantin (UniProt accession number: P84072.1), trypsin inhibitor BGIT (UniProt accession number: 1VBW_A), and MC2-1-5 [7] protein were selected and downloaded from the UniProt database (*https://www.uniprot.org/*) (Consortium, 2015).

### *In silico* peptide designing and screening

Peptide Cutter Server (*http://www.expasy.ch/tools/peptidecutter/*) was used to digest or cut the hypoglycemic protein sequence into peptides using trypsin and pepsin enzymes [17]. Peptides were screened further using Peptide Ranker tool (http://distilldeep.ucd.ie/PeptideRanker/) to predict their bioactive potential. This tool assigns a score to peptides that indicate the likelihood of the peptide being bioactive. Peptides showing scores more than 0.5 are likely to be bioactive and were selected in this study for subsequent analyses. In order to examine the toxicity of peptides ToxinPred tool (http://crdd.osdd.net/raghava/toxinpred/) was used [18]. Half-life of peptides was evaluated using the HLP server (*http://crdd.osdd.net/raghava/hlp/pep_both.htm*) with an SVM-based model in the intestinal conditions. The peptides with high stability and high half-life were selected for further analyses [17].

### Receptor and ligand preparation

An online tool Pepfold 3 was used to predict three-dimensional (3D) structures of selected peptides and were saved in PDB format (https://mobyle.rpbs.univ-paris-diderot.fr/cgi-bin/portal.py#forms::PEP-FOLD3) [19]. Ten proteins were selected based on their antidiabetic effect reported in literature. The 3D structures of selected proteins including dipeptidyl peptidases IV (PDB ID: 1NU6), insulin-like growth factor 1 receptor (PDB ID: 1K3A), glucose transporter GLUT1 (PDB ID: 4PYP), peroxisome proliferator-activated receptor gamma (PDB:4 CI5), maltase-glucoamylase (PDB: 3TOP), adiponectin (PDB: 4DOU), sodium–glucose co-transporter 2 (SGLT-2) (PDB ID:7VSI), c-Jun N-terminal kinase 1 (JNK1) (PDB:3ELJ), aldose reductase (PDB: 3RX2), glucokinase (PDB ID: 1V4S), and pancreatic alpha-amylase (PDB: 1B2Y) as target or receptor proteins involved in diabetes were downloaded from the Protein Data Bank (PDB-RCSB) (*https://www.rcsb.org*) in PDB format [20]. The receptor proteins were introduced to Molecular Operating Environment (MOE) software and were prepared for docking by eliminating all other chains except the single alpha chain. Hydrogen atoms were added, and 3D protonation and energy minimization steps were performed.

### Protein-peptide docking study

The molecular docking was performed using haddock 2.4 an online tool to predict how a peptide and a protein interact with each other (https://rascar.science.uu.nl/haddock2.4/), which results in more accurate interaction models [21]. Docking complexes for each protein-peptide were analyzed on the basis of their HADDOCK scores. A highly negative HADDOCK score shows a better binding affinity. The PyMOL Molecular Graphics System’s educational version was used to visualize and demonstrate the interactions among receptor proteins and hypoglycemic peptides within the active site of the selected receptor protein [22]. Additionally, the interactions between the amino acid residues in the docked complex were verified using PDBsum (https://www.ebi.ac.uk/thornton-srv/databases/pdbsum/) [23].

### Molecular dynamics simulation

Desmond (a Package of Schrödinger LLC) was used to perform MD simulations for 100 nanoseconds to check the stability of the peptide-protein complex. The initial stages of peptide-protein complex were obtained from molecular docking for MD simulation. MD simulation helps predicting the binding status of the complex in the physiological environment [24]. The Protein Preparation Wizard or Maestro was used to process peptide-protein complex including the optimization and minimization of the complex. The System Builder tool was employed to prepare all systems and the Solvent Model with an orthorhombic box was selected as Transferable Intermolecular Interaction Potential 3 Points. The force field of OPLS_2005 was used in the simulation and the models were made neutral by addition of counter ions [25]. Sodium chloride (0.15 M) was added to mimic the physiological conditions. The models were relaxed before the simulation and the isothermal-isobaric (NPT) ensemble was selected with a temperature of 310 K and pressure of 1 atm for the whole simulation time. For analysis, the trajectories were saved after every 100 ps and the simulation stability was checked by calculating the RMSD of the peptide and the protein over time. The Desmond simulation trajectories were studied to calculate RMSD, root mean square fluctuation (RMSF), and peptide-protein contacts [26].

## Results and discussion

The selected hypoglycemic proteins including Cq-IMP, AdMc1, polypeptide-P, prolamin binding factor, MC6, charantin, trypsin inhibitor BGIT, and MC2-1-5 were digested using Peptide Cutter Server to generate peptides using trypsin and pepsin enzymes. The generated peptides are shown in S1 Table. The peptides were characterized for their bioactive potential, bioactivity, stability, and half-life and the results of best selected peptides are shown in Table 1. The detailed characteristics of all generated peptides are given in S2 Table.

**Table 1:** Screening of top eight selected peptides using Peptide Ranker and ToxinPred tools.

Peptides number and sequence	Peptide Ranker score	Svm score	Prediction	Hydrophobicity	Charge	PI	MW	Half-life (sec)	Stability
P1 SSWPQL	0.833612	−1.00	Non-Toxin	−0.06	0.00		716.87	1.239	High
P2 CYYNNYSMSL	0.602973	−0.49	Non-Toxin	−0.09	0.00	5.84	1257.53	0.827	Normal
P3 NVPIGGGCRKPK	0.512157	−0.57	Non-Toxin	−0.25	3.00	10.07	1225.65	1.674	High
P4 PTFMSGG	0.621438	−0.86	Non-Toxin	0.10	0.00	5.88	695.88	1.452	High
P5 GGAGG	0.52423	−0.81	Non-Toxin	0.18	0.00	5.88	317.37	1.366	High
P6 LGPNNGYYYGGHANGSSIGM	0.637122	−0.39	Non-Toxin	−0.01	0.50	7.08	2029.48	2.489	High
P7 GIPSPMQQHGGL	0.808791	−1.27	Non-Toxin	−0.01	0.50	7.10	1221.59	0.000	Low
P8 QWQPHVGNGGGGVVG	0.525456	−1.15	Non-Toxin	0.03	0.50	7.10	1448.81	1.310	High

PI: Isoelectric Point, MW: Molecular Weight.

Protein-protein interactions (PPIs) cause two or more protein molecules to combine to form complexes and aggregates. They are essential for numerous biological activities, including viral self-assembly, gene expression and regulation, cell signaling, and antibody–antigen interactions [27]. However, PPIs are regulated in different ways and include more diverse processes. One must recognize different interactions and identify the consequences of the interactions in order to gain a more precise grasp of their significance in the cell [28]. In the current study, to identify possible interactions between hypoglycemic peptides and diabetic receptor proteins, High Ambiguity Driven DOCKing (HADDOCK v2.4), a flexible docking server was used to model complex bimolecular interactions.

In the current study, among eight selected hypoglycemic peptides only three peptides showed best HADDOCK score and binding interactions with the selected receptor proteins. Complexes with the lowest binding energy were considered most stable (Table 2). Peptide P6 showed strong interactions and hydrogen bonding with receptor proteins 1b2y, 3rx2, 1k3a, 1v4s, and 4 ci5, peptide P2 showed strong interactions with active site residues of receptor proteins 3top, 4dou, and 4pyp. The peptides P5 and P6 showed similar haddock scores with receptor protein 3elj with different Z-scores. The HADDOCK score combines electrostatic energy, van der Waals energy, desolvation energy, and restraints violation penalties to validate protein-peptide poses. It also prioritizes clusters of those structures which satisfy the experimental or in silico derived restraints such as mutation or interaction data [29]. Similarly, the Z-score in protein-peptide docking is used to quantify how a cluster’s HADDOCK score deviated from the average. More negative Z-score indicates a better docking solutions relative to others [30].

**Table 2:** Binding scores (in kcal/mol) and Z-scores (in parenthesis) of top eight hypoglycemic peptides with selected receptor proteins.

Peptide	1B2Y	3RX2	1K3A	1NU6	1V4S	3ELJ	3TOP	4 CI5	4DOU	4PYP
P1	−74.2 + /- 1.0 (−1.4)	−62.5 + /- 5.0 (−2.0)	−48.4 + /- 1.2 (−2.4)	−44.6 + /- 7.0 (−1.9)	−59.8 + /- 2.1 (−1.4)	−58.0 + /- 0.6 (−1.0)	−71.1 + /- 0.6 (−1.9)	−59.6 + /- 2.0 (−2.0)	−36.6 + /- 7.0 (−2.4)	−72.0 + /- 4.4 (−1.5)
P2	−96.6 + /- 1.6 (−1.2)	−72.9 + /- 3.4 (−1.7)	−66.6 + /- 9.1 (−1.2)	−63.3 + /- 4.3 (−1.6)	−64.8 + /- 0.7 (−1.8)	−77.8 + /- 3.4 (−1.8)	−108.7 + /- 9.3 (−1.4)	−72.0 + /- 2.4 (−1.4)	−49.8 + /- 7.1 (−1.8)	−85.3 + /- 1.4 (−1.8)
P3	−93.2 + /- 2.7 (−1.7)	−52.9 + /- 2.4 (−1.6)	−55.7 + /- 3.5 (−2.1)	−59.3 + /- 4.8 (−1.9)	−61.0 + /- 5.6 (−1.3)	−45.8 + /- 1.7 (−1.6)	−85.9 + /- 5.3 (−1.9)	−58.5 + /- 5.0 (−1.9)	−38.5 + /- 6.2 (−1.3)	−59.5 + /- 2.5 (−1.5)
P4	−77.8 + /- 1.0 (−1.5)	−57.9 + /- 3.4 (−1.9)	−38.7 + /- 2.3 (−2.1)	−47.8 + /- 3.4 (−2.1)	−60.8 + /- 2.8 (−1.2)	−52.6 + /- 0.6 (−1.2)	−76.3 + /- 1.4 (−1.7)	−58.3 + /- 0.3 (−2.0)	−38.5 + /- 5.8 (−2.1)	−72.2 + /- 1.3 (−1.7)
P5	−36.3 + /- 0.5 (−2.1)	−26.5 + /- 3.6 (−1.5)	−3.0 + /- 2.0 (−1.8)	−15.9 + /- 7.7 (−1.3)	−33.7 + /- 2.0 (−1.4)	−92.1 + /- 4.1 (−1.1)	−46.4 + /- 2.2 (−1.8)	−39.5 + /- 2.2 (−1.2)	−11.5 + /- 6.2 (−2.0)	−43.3 + /- 1.3 (−1.5)
P6	−121.3 + /- 3.1 (−2.3)	−84.0 + /- 5.7 (−1.7)	−107.5 + /- 5.4 (−2.8)	−78.3 + /- 3.1 (−1.7)	−90.1 + /- 1.2 (−1.6)	−92.1 + /- 4.1 (−1.7)	−93.2 + /- 9.1 (−1.0)	−78.9 + /- 5.4 (−1.7)	−36.1 + /- 6.8 (−1.5)	−78.9 + /- 0.4 (−1.2)
P7	−75.0 + /- 3.6 (−1.0)	−58.0 + /- 1.3 (−1.3)	−55.0 + /- 7.6 (−1.7)	−55.1 + /- 1.6 (−2.0)	−68.9 + /- 0.6 (−1.5)	−55.7 + /- 1.7 (−1.7)	−81.7 + /- 2.4 (−1.2)	−46.3 + /- 7.7 (−1.8)	−21.9 + /- 2.0 (−2.0)	−82.2 + /- 3.7 (−2.2)
P8	−95.9 + /- 1.9 (−1.5)	−59.3 + /- 2.3 (−1.6)	−80.8 + /- 3.0 (−1.7)	−70.0 + /- 3.3 (−1.9)	−66.3 + /- 3.0 (−2.0)	−70.1 + /- 1.5 (−1.6)	−86.8 + /- 1.6 (−2.0)	−55.7 + /- 3.1 (−2.0)	−35.7 + /- 7.7 (−1.9)	−77.1 + /- 12.0 (−1.7)

### Interaction between HGPs and human pancreatic α-amylase

**Fig 1:**
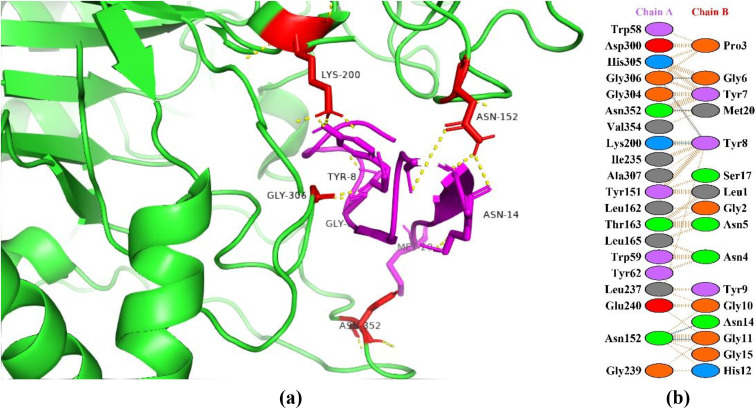
Protein-peptide interactions between P6 and pancreatic α-amylase. (a) Receptor protein is represented in green with red interacting residues, and peptide P6 is shown in magenta with interacting residues. (b) Residual interactions between P6 and protein complex; hydrogen bonds are represented in blue, salt bridges are shown by red colored lines. Other amino acids representative properties are shown by different colors (i.e., aliphatic: grey, positive: blue, negative: red, Cys: yellow, aromatic: pink, Pro and Gly: orange, and neutral: green).

The human pancreatic α-amylase (HPA) is primarily responsible for starch digestion by hydrolyzing the α-1,4-glycosidic bond found in glycogen, amylose, starch, dextrin, and amylopectin [31]. Over conversion can raise blood sugar levels in tissues and in certain situations and hyperglycemia can occur as a result of α-amylase over activation and insulin insufficiency [32]. Hence, HPA inhibition ability to delay the digestion of starch is crucial for managing postprandial hyperglycemia in type II diabetes mellitus. Inhibiting HPA in small intestine slows down the rate at which starch hydrolyzes and delays the digestion process. One of the most efficient methods for reducing postprandial hyperglycemia is the spreading of the digestive process, which lowers the quantity of glucose produced and released in bloodstreams [33]. In this study, peptide P6 showed the best haddock score of −121.3 + /- 3.1 kcal/mol forming interactions with the active site amino acids of α-amylase making seven hydrogen bonds (Fig 1). The protein is highlighted as green color with red highlighted sticks as interacting amino acids and peptide in magenta color showing interactions in yellow. In a study, interactions of 93 compounds were checked from commercial and traditional rice varieties with the catalytic site of α-amylase. Two compounds, i.e., triacontane (with HADDOCK score of −9.98 kcal/mol) and β-D-glucopyranoside methyl (with HADDOCK score of −9.58 kcal/mol) exhibited the lowest binding energies [34]. In another study, Xavier et al. [35] investigated the *in vitro* and *in silico* pharmacological properties of *Oxystelma esculentum* and reported that the ligand solanesol showed better binding affinity with a docking score of −7.3 kcal/mol towards human pancreatic α-amylase.

### Interactions between HGPs and insulin-like growth factor 1 receptor

**Fig 2:**
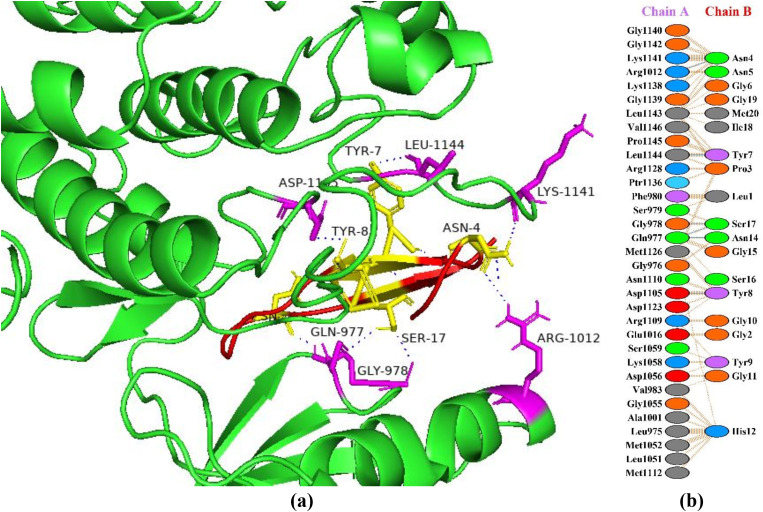
Protein-peptide interactions between P6 and insulin-like growth factor 1 receptor. (a) Receptor protein is represented in green with interacting residues in magenta. The peptide P6 is shown in red with its interacting residues in yellow. (b) Residual interactions between P6 and protein complex; hydrogen bonds are represented in blue, salt bridges are shown by red colored lines. Other amino acids representative properties are shown by different color (i.e., aliphatic: grey, positive: blue, negative: red, Cys: yellow, aromatic: pink, Pro and Gly: orange, and neutral: green).

Insulin-like growth factor 1 (IGF-1) belongs to a family of insulin related peptides which play important roles in metabolism, cell differentiation, and growth and development. Brain, bones, liver, and testes are among the body systems that have been shown to contain the IGF-1 receptor. This implies that IGF plays a significant paracrine and endocrine role [36]. The primary mechanism of action of IGF-1, IGF-2, and insulin is through tyrosine-kinase-linked receptors specifically the insulin receptor (IR) and the IGF-1 receptor (IGF1R). The IR has a strong affinity for insulin, while the IGF1R has a strong affinity for IGF-1 and IGF-2 [37]. Compared to IGFs, insulin has a lower affinity for the IGF-1 receptor, and IGF binds to insulin receptor to initiate body’s blood glucose level reduction [36]. As both receptors have a lot of structural and functional similarities, it is also possible for these two receptors to combine to form heterodimers that bind both ligands. When the receptors are bound, tyrosine and serine kinase cascades are triggered to promote growth or metabolism [37]. The A and B isoforms of insulin receptor interact with IGFs. To balance the metabolism of fat, protein, and carbohydrates these interactions mediate signals in different tissues. In this study, the peptide P6 showed the best binding pattern with insulin-like growth factor 1 receptor with a docking score of −107.5 + /- 5.4 kcal/mol with seven hydrogen bonds (Fig 2). In a study, Singh et al. [38] predicted a strong binding interaction of the ligand (with docking score of –5.07 kcal/mol) and with estimated RMSD of 0.00. In another study, Damián-Medina et al. [39] analyzed antidiabetic potential of phenolic compounds from blue corn and black beans and found petunidin 3-O-glucoside with the best binding energy of −5.47 kcal/mol followed by cyanidin 3-glucoside with a binding energy −5.16 kcal/mol.

### Interactions between HGPs and aldose reductase

**Fig 3:**
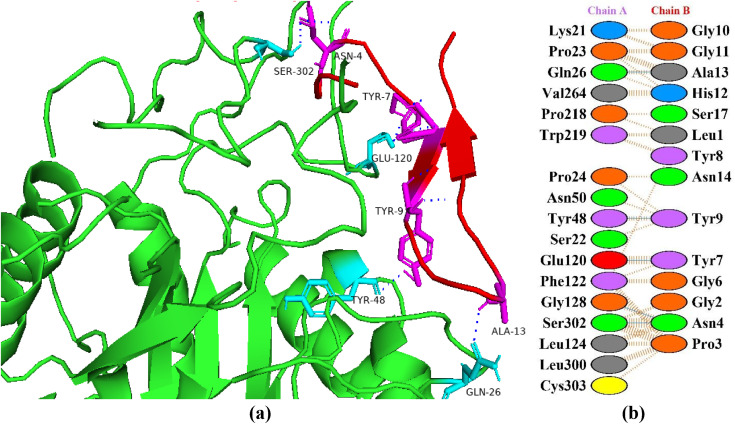
Protein-peptide interactions between P6 and aldose reductase. (a) Receptor protein is represented in green with cyan interacting residues. The peptide P6 is shown in red with its interacting residues in magenta. (b) Residual interactions between P6 and protein complex. Hydrogen bonds are represented in blue, salt bridges are shown by red colored lines. Other amino acids representative properties are shown by different colors (i.e., aliphatic: grey, positive: blue, negative: red, Cys: yellow, aromatic: pink, Pro and Gly: orange, and neutral: green).

Aldose reductase belongs to a superfamily of aldo–keto reductases and is a monomeric reduced NADPH dependent cytosolic enzyme. Aldose reductase coverts glucose into sorbitol and other alcoholic sugars via pylol pathway utilizing NADPH as a cofactor and is also a rate limiting enzyme [40]. Aldose reductase is activated by excessive glucose saturation during hyperglycemia. Diabetic complications such as diabetic retinopathy and diabetic nephropathy are indirectly brought on by this fact (pathways/transformation). Aldose reductase inhibitors, which are rarely available, will therefore lower the glucose flux and are essential for the treatment of diabetes [41]. In this study, aldose reductase was focused in protein-protein docking to identify potential hypoglycemic peptide(s). Among the selected peptides, P6 was found to be the top peptide with haddock score of −84.0 + /- 5.7 kcal/mol and with eight hydrogen bonds with aldose reductase (Fig 3). In a study, Reddy et al. (2023) docked thirteen phytocompounds against aldose reductase as a receptor protein and found strong binding affinities with rhynchosin (with docking score of −10.0 kcal/mol) and biochanin (with docking score of −9.9 kcal/mol). In another study, based on network pharmacology of phytocompounds having antidiabetic attributes, it was reported that the compound chicoric acid showed the best binding affinity with docking score of −11 kcal/mol and with ten hydrogen bonds with aldose reductase receptor [42].

### Interactions between HGPs and dipeptidyl peptidase-4

**Fig 4:**
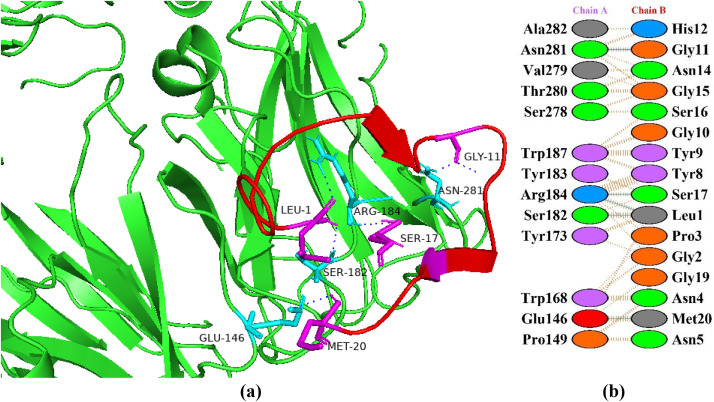
Protein-peptide interactions between P6 and dipeptidyl peptidases-4. (a) Receptor protein is represented in green with cyan interacting residues. The peptide P6 is shown in red with its interacting residues in magenta. (b) Residual interactions between P6 and protein complex. Hydrogen bonds are represented in blue, salt bridges are shown by red colored lines. Other amino acids representative properties are shown by different colors (i.e., aliphatic: grey, positive: blue, negative: red, Cys: yellow, aromatic: pink, Pro and Gly: orange, and neutral: green).

Dipeptidyl peptidase-4 (DPP-4) is a multitasking protein which deploys its biological activity via pleotropic actions such as protease activity, association with adenosine deaminase, extracellular matrix interaction, cell surface co-receptor activity mediating viral entry as well as regulating signal transduction within the cell coupled to control cell proliferation and migration control [43]. DPP-4 deactivates two main incretins, i.e., glucagon-like peptide-1 (GLP-1) and gastric inhibitory polypeptide (also known as glucose-dependent insulinotropic polypeptide (GIP)), which are necessary to promote regular insulin secretion from the pancreatic beta cells. Consequently, DPP-4 needs to be inhibited in order for incretins to function properly [44]. In the current study, the peptide P6 showed the best binding patterns with dipeptidyl peptidases-4 with a docking score of −78.3 + /- 3.1 kcal/mol and five hydrogen bonds (Fig 4). In a study, Nongonierma et al. [45] reported that a tri-peptide (Trp-Trp-Trp) among eight thousand tri-peptides which were derived from milk protein showed the best docking score of −10.8 kcal/mol. In another study, Cian et al. [46] identified a peptide (i.e., SLAVSVH) which showed a binding energies of −7.65 kcal/mol and −1392 ± 4 kcal/mol via autodock and flexpepdock, respectively.

### Interactions between HGPs and glucokinase

**Fig 5:**
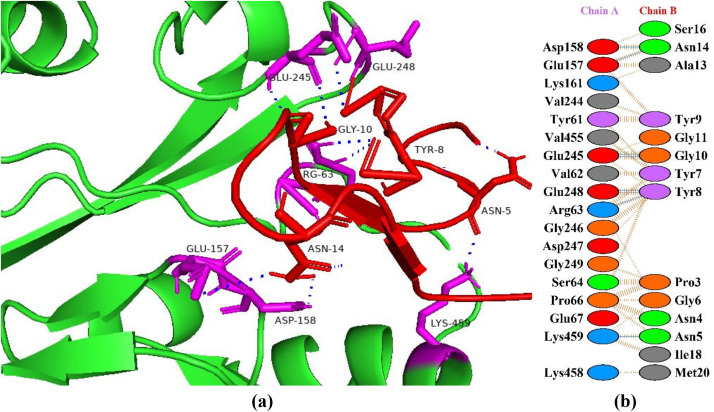
Protein-peptide interactions between P6 and glucokinase. (a) Receptor protein is represented in green with magenta interacting residues. The peptide P6 is shown in red with its interacting residues. (b) Residual interactions between P6 and protein complex. Hydrogen bonds are represented in blue, salt bridges are shown by red colored lines. Other amino acids representative properties are shown by different colors (i.e., aliphatic: grey, positive: blue, negative: red, Cys: yellow, aromatic: pink, Pro and Gly: orange, and neutral: green).

A special hexokinase enzyme isoform that converts glucose to glucose-6 phosphate is called glucosekinase [47]. Glucokinase is a key player in glucose homeostasis; specifically, it stimulates the production of glycogen in liver and aids the release of insulin in response to glucose in pancreas [48]. In addition to controlling liver’s conversion of glucose into glycogen and hepatic glucose production, glucokinase acts as a glucose sensor for insulin-producing pancreatic islet β-cells [49]. Glucokinase is regarded as an appealing therapeutic target in diabetes because of this activity [48]. In the current study, the peptide P6 revealed a haddock score of −90.1 + /- 1.2 kcal/mol with eight hydrogen bonds (Fig 5). In a study, Rathinavel et al. [50] observed β-amyrin with a strong binding affinity (docking score of −8.2 kcal/mol) against glucokinase. Similarly, Khan et al. [47] reported different phytocompounds including apigenin, genkwanin, and swertiamarin of *Enicostemma littorale* which exhibited binding energies of −8.7 kcal/mol, −7.5 kcal/mol, and −8.3 kcal/mol, respectively and could be used to regulate glucokinase.

### Interactions between HGPs and Jun N-terminal kinase 1

**Fig 6:**
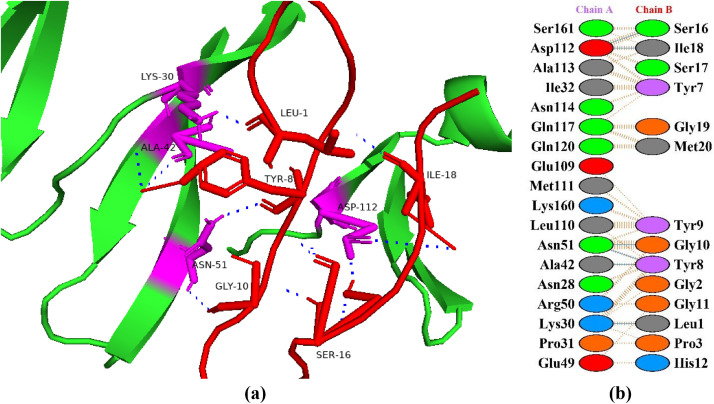
Protein-peptide interactions between P6 and Jun N-terminal kinase1. (a) Receptor protein is represented in green with magenta interacting residues. The peptide P6 is shown in red with its interacting residues. (b) Residual interactions between P6 and protein complex. Hydrogen bonds are represented in blue, salt bridges are shown by red colored lines. Other amino acids representative properties are shown by different colors (i.e., aliphatic: grey, positive: blue, negative: red, Cys: yellow, aromatic: pink, Pro and Gly: orange, and neutral: green).

**Fig 7:**
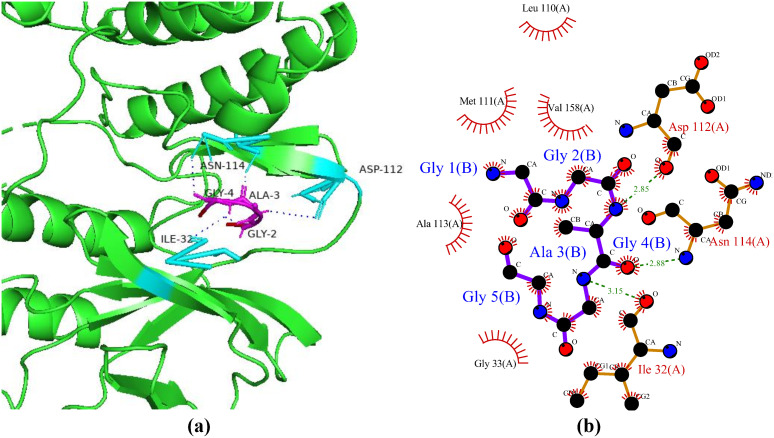
Protein-peptide interactions between P5 and Jun N-terminal kinase1. (a) Receptor protein is represented in green with cyan interacting residues. The peptide P5 is shown in magenta with its interacting residues. (b) Residual interactions between P5 and protein complex. Hydrogen bonds are represented in green color.

JNK1, or Jun N-terminal kinase 1 is a member of the mitogen-activated protein kinase superfamily [51]. In macrophages, JNK1 is essential for the emergence of insulin resistance. Therefore, JNK1 may affect macrophage infiltration of adipose tissues and change the production of inflammatory cytokines (such as tumor necrosis factor), which are thought to be mediators of insulin resistance [52]. Obesity and type 2 diabetes are linked to JNK1 pathway activation and the absence of JNK1 provides significant protection against insulin resistance brought on by obesity. These findings clearly imply that JNK1 is essential for the development of insulin resistance in type 2 diabetes. JNK1 pathway suppression results in the decrease in the expression of key gluconeogenic enzymes and reduction of glucose production in liver. The absence of JNK1 increases the insulin receptor signaling capacity by its effect on IRS-1 phosphorylation. Taken together, obesity and type 2 diabetes mellitus are associated with the activation of JNK1 pathway, while its absence causes substantial protection from the induction of insulin resistance from obesity. These finding suggest that JNK1 is involved in insulin resistance, which is a type 2 diabetes mellitus. Thus, the selective inhibition of JNK1 activity is probably a therapeutic target of type 2 diabetes, insulin resistance, and obesity [53]. In this study, the peptides P5 and P6 showed same haddock score (i.e., −92.1 + /- 4.1 kcal/mol). The peptide P6 exhibited six H-bonds with JNK1 (Fig 6), while the peptide P5 exhibited three H-bonds with JNK1 receptor (Fig 7). In a study, Mandar et al. [54] explored *Tinospora cordifolia* phytoconstituents through an *in silico* study and found berberine to be the best compound with strong binding affinity of −9.5 kcal/mol against JNK1 receptor protein. Similarly, Kurniawan et al. [55] studied algal peptides and found peptide P13 with the highest binding affinity −8.8 kcal/mol against JNK1, showing considerable interacting potential. The P13 energy was significantly higher than the control drugs Metformin (binding affinity of −4.4 kcal/mol) and Pioglitazone (binding affinity of −7.5 kcal/mol).

### Interactions between HGPs and maltase-glucoamylase

**Fig 8:**
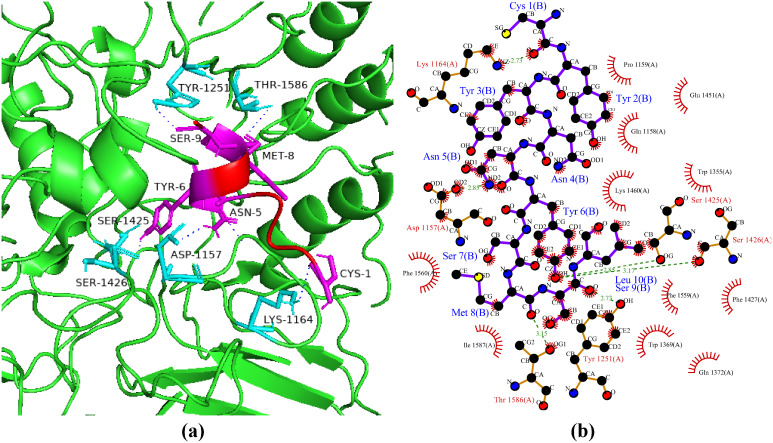
Protein-peptide interactions between P2 and maltase-glucoamylase. (a) Receptor protein is represented in green with cyan interacting residues. Peptide P2 is shown in red with its interacting residues in magenta. (b) Residual interactions between P2 and receptor complex in which hydrogen bond are represented as green dotted lines.

Endohydrolases and exohydrolases are enzymes that humans need to fully digest starch and convert it into glucose. The endohydrolases are known as salivary and pancreatic α-amylases and break down the internal alpha 1–4 bonds of starch into linear, shorter, and branching chains of dextrin. Then, two small-intestinal brush-border exohydrolases (i.e., sucrose isomaltase and maltase glucoamylase) further hydrolyze the resulting mixture of dextrin at the non-reducing ends to produce glucose [56]. In the human small intestine, maltase-glucoamylase (MGA) is a particular type of alpha-glucosidase digestive enzyme that breaks down carbohydrates into glucose in conjunction with sucrose-isomaltase and α-amylase [57]. Human pancreatic α-amylase (HPA) activity and MGA inhibition have a major impact on blood glucose regulation, making them ideal targets in the treatment of diabetes mellitus type 2 and obesity [56]. In the current study, the peptide P2 showed the best binding pattern with a haddock score of −108.7 + /- 9.3 kcal/mol with six hydrogen bonds with maltase-glucoamylase (Fig 8). Guan et al. [58] reported in a study conducted on dipeptides that peptides TW and WA exhibited strong binding energies of −229.93 ± 38.6 kcal/mol and −112.53 ± 38.68 kcal/mol, respectively. In another study, Abdulhaniff et al. [59] revealed inhibitory activity of rhamnosyl hexosyl methyl quercetin (RHMQ), p-coumaryl-O-16-hydroxy palmitic acid (PCHP), and spirostanol against MGA when docked these showed binding affinities of −12.88 kcal/mol, −8.59 kcal/mol, and −7.87 kcal/mol, respectively.

### Interactions between HGPs and PPAR-γ

**Fig 9:**
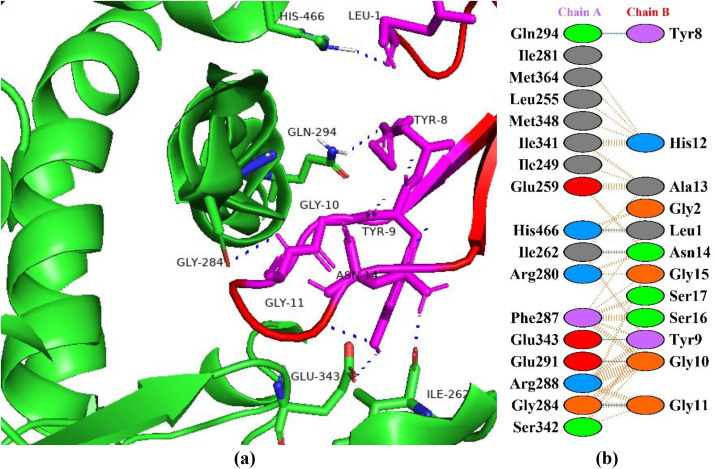
Protein-peptide interactions between P6 and PPAR-γ. (a) Receptor protein is represented in green with its interacting residues. The peptide P6 is shown in red with its interacting residues in magenta. (b) Residual interactions between P6 and PPAR-γ complex. Hydrogen bonds are represented in blue, salt bridges are shown by red colored lines. Other amino acids representative properties are shown by different colors (i.e., aliphatic: grey, positive: blue, negative: red, Cys: yellow, aromatic: pink, Pro and Gly: orange, and neutral: green).

PPAR-γ is a member of superfamily receptors that respond to small lipophilic ligands by controlling the transcription of genes [60]. These ligands activate PPAR-γ and speed up the process by which fibroblasts differentiate into adipocytes and increase the expression of lipoprotein lipase, GLUT4, and of insulin receptor substrates 1 and 2 (IRS-1 and IRS-2) in skeletal muscle and adipose tissues and decrease TAG blood concentrations [61]. In the current study, the peptide P6 showed haddock score of −78.9 + /- 5.4 kcal/mol with the receptor PPAR-γ with six hydrogen bonds (Fig 9). In a study, Yadav et al. [62] conducted molecular docking and MD simulation study on *Costus igneus* novel derivatives against diabetes and revealed β-sitosterol as the best ligand with binding affinity of −12.29 kcal/mol against PPAR-γ. Similarly in another study, Mandar et al. [54] reported isocolumbin with the highest binding affinity of −10.1 kcal/mol against PPAR-γ showing one hydrogen bond with amino acid Arg288.

### Interactions between HGPs and adiponectin

**Fig 10:**
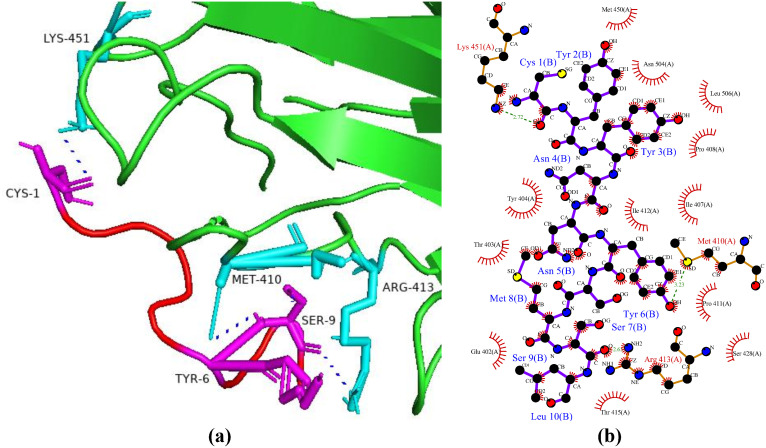
Protein-peptide interactions between P2 and adiponectin. (a) Receptor protein is represented in green with cyan interacting residues. The peptide P2 is shown in red with its interacting residues in magenta. (b) Hydrogen bond residual interactions between P2 and adiponectin complex are represented as green dotted lines.

Adiponectin is a member of collagen-like proteins which is secreted by adipocytes and has an action as a hormone with insulin sensitizing and anti-inflammatory properties. Animals studies and human metabolic studies revealed different mechanisms by which adiponectin decreases the risk of diabetes by involving liver gluconeogenic suppression, simulation of hepatic and muscle fatty acid oxidation, glucose uptake, and simulating insulin secretion. These effects might partially be mediated by adiponectin’s stimulatory effects on the signaling pathways for peroxisome proliferator-activated receptor and adenosine monophosphate-activated protein kinase [63]. The locus for diabetes susceptibilty have been mapped on chromosome 3q27 where the gene for adiponectin is located. As indicated by both genetic and functional data, adiponectin may play a role in the pathophysiology of type 2 diabetes. It has also been demonstrated that in an animal model of diabetes, lower levels of adiponectin occur before the disease manifests. Conversely, elevated levels of adiponectin may delay the development of type 2 diabetes [64]. In this study, the peptide P2 showed haddock score of −49.8 + /- 7.1 kcal/mol with three hydrogen bond interactions (Fig 10). In a study, Mandar et al. [54] reported in a computational study that isocolumbin had the highest binding energy of −9.0 kcal/mol against receptor adiponectin. Similarly, in another study, Khanal et al. [65] reported that sitagliptin had the best binding score of –7.1 kcal/mol against this receptor protein.

### Interactions between HGPs and GLUT1

**Fig 11:**
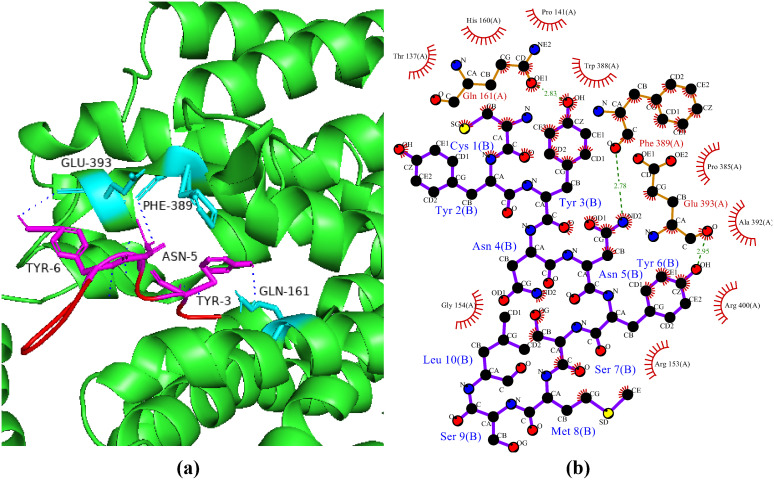
Protein-peptide interactions between P2 and GLUT1. (a) Receptor protein is represented in green with cyan interacting residues. The peptide P2 is shown in red with its interacting residues in magenta. (b) Hydrogen bond residual interactions between P2 and GLUT1 complex are represented as green dotted lines.

The GLUT protein family isoforms are the main transporters by which glucose moves throughout the body [66]. GLUT1 a hydrophobic integral membrane protein made up of 492 amino acids which facilitates the movement of ascorbic acid, glucose, galactose, mannose, and glucosamine [67]. The most well-known member of the family is GLUT1, which is found everywhere and works with other GLUTs to transport into most of the tissues. However, it is primarily located at the plasma membrane and is specifically in charge of transportation across the blood–retinal barrier (BRB). GLUT1 transport activity is regulated differently other than its localization in comparison to GLUT4 whose activity is regulated by blood glucose level. Blocking GLUT1 would protect against diabetes and its other complications because excessive GLUT1 transport causes high glucose levels in diabetics’ retinas [68]. In the current study, the peptide P2 showed the best haddock score of −85.3 + /- 1.4 kcal/mol with three interacting hydrogen bonds (Fig 11). In a study Chaudhary et al. [69] reported that β-carotene with docking score of −11.1 kcal/mol and zeaxanthin with docking score of −10.9 kcal/mol had the strongest binding affinities in the evaluation of *Stigma maydis* antidiabetic potential. In another study, Rathore et al. [70] reported gymnemagenin with docking score of −7.86 kcal/mol and snitaglipti with docking score of −8.04 kcal/mol had the strongest binding energies against GLUT1 receptor protein.

### Validation through MD Simulation

Based on binding scores of peptide-protein docking, two peptides (i.e., P2 and P6) selected for molecular dynamics simulation study against different proteins. The binding stability of P2 with protein maltase-glucoamylase and of P6 with proteins pancreatic alpha-amylase, insulin-like growth factor 1 receptor, and JNK1 were validated through MD simulation. Atomic movements are typically computed over time using MD simulations. Peptide binding status was predicted through MD simulation in the physiological environment [71].

**Fig 12:**
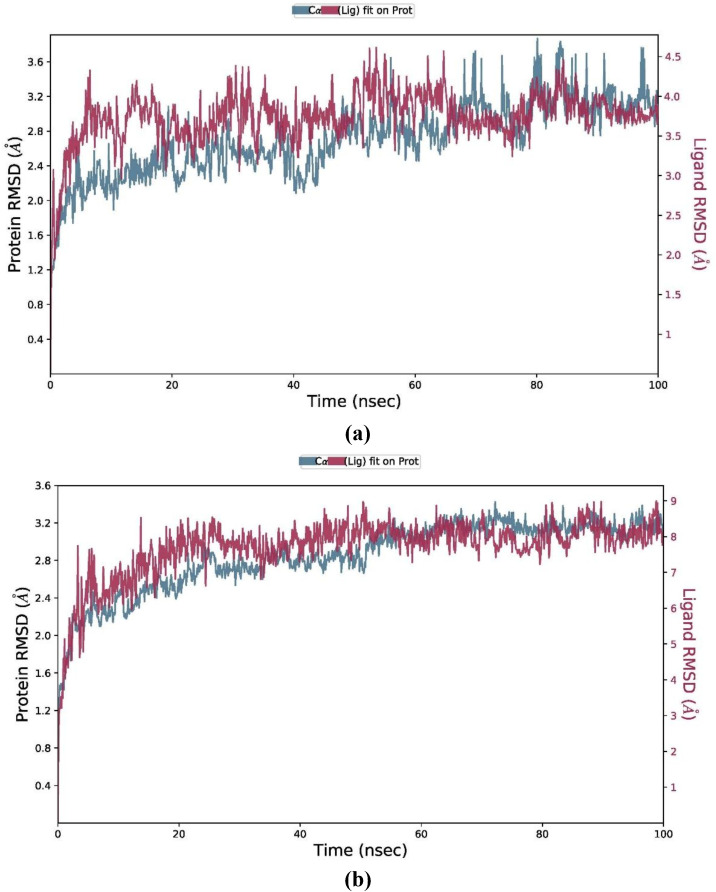
MD simulation showing RMSD trajectories. The MD simulation of the docked complex of (a) P6- insulin-like growth factor 1 receptor and (b) P2- maltase-glucoamylase.

In Fig 12a (P6-1K3A), the RMSD of both protein and the peptide increased rapidly from 0–10 ns, which is showing the initial relaxation and adaptation to the conditions of MD simulation. From 10–60 ns, both protein and peptide RMSD were observed to be fluctuated but generally stabilized and showing that the system was equilibrated. The RMSD of the protein got stabilized from 2.2–2.8 Å, while that of the peptide was found to be slightly higher and fluctuated from 2.5–3.2 Å. Although the RMSD values of protein and peptide showed continued fluctuations but without any dramatic increases or decreases, which has suggested that the system remained stable throughout simulation time. Some occasional spikes were observed, which might be due to transient conformational changes or peptide repositioning, but the overall trend was found to be stable over the 100 ns simulation.

Fig 12b (P2-3TOP) is showing RMSD plot of protein and peptide P2 over a 100 ns simulation time. The RMSD of protein and peptide from 0-20 ns was increased rapidly showing conformational changes. The RMSD values from 20-100 ns were appeared to plateau which indicated that the system has achieved the equilibrium state. The RMSD of the protein fluctuated around 2.8 Å and that of the peptide fluctuated around 8 Å, which showed that both the protein and the peptide were exploring different conformations but the overall structure was found to be relatively stable. The RMSD of the peptide was higher than the protein, which is indicating that the peptide has more conformational flexibility. Overall, the relative sable RMSD values of the complex after the initial phase has shown that the simulation was reached to a stable state and the complex was maintained through the MD simulation.

Similarly, the RMSD analyses of P6 with JNK1 and pancreatic alpha-amylase over MD simulation of 100 ns is shown in S1 Fig. RMSD value is used to validate structural stability and conformational changes of peptide and the protein during the simulation time. It has been shown that protein remained relatively stable throughout the simulation especially after the 1^st^ 20 ns but the peptide showed instability and probable unbinding events at different intervals especially before 20 ns and between 30–40 ns of the simulation (S1a Fig). The peptide got bound more consistently in the 2^nd^ half of the MD simulation especially after 55 ns, which is suggesting a possible conformational accommodation by the protein its improved binding mode.

In P6-pancreatic alpha-amylase complex, the RMSD of the protein was found to be 0.5 Å in the start, which was continuously increased and plateaued 1.5–2.0 Å after 70 ns and showing a gradual conformational adjustment (S1b Fig). In the RMSD graph, no sharp spikes were observed which showed an overall stable trajectory with progressive structural adaptation which might be due to the interactions with the peptide. The RMSD plot of the peptide was started around 1 Å and increased to 11.5 Å. Early fluctuations from 0–20 ns are suggesting instability as the peptide might be exploring multiple orientations within the binding pocket. After 20–30 ns, the peptide RMSD was found to be continuously increased which is showing that the peptide might not be remained stably bound. The RMSD values of 8–10 Å are suggesting that the peptide could unbind or drift significantly from the original docked pose.

**Fig 13:**
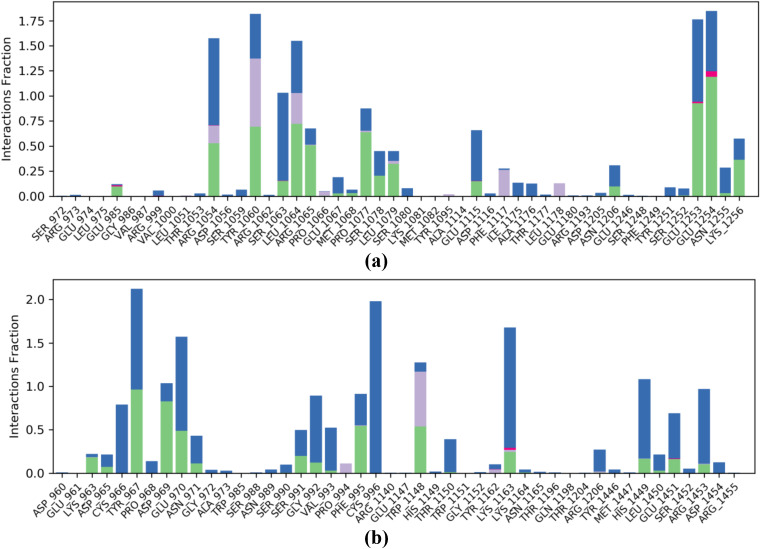
Histograms of protein-peptide contact. (a) Insulin-like growth factor 1 receptor complexed with P6; (b) maltase-glucoamylase complexed with P2.

The Protein Ligand Interaction Fingerprints (PLIF) of the insulin-like growth factor 1 receptor complexed with P6 before and after MD simulation revealed that majorly the residues Arg1054, Tyr1060, Leu1064, Arg1065, Pro1077, Ser1078, Leu1079, Glu1253, Glu1254, and Lys1256 were involved in stabilizing the complex through hydrogen bonds with the P6. Similarly, the residues Arg1054, Tyr1060, Leu1064, and Phe1117 were also involved in hydrophobic interactions with the peptide P6 (Fig 13a). From the PLIF diagram of maltase-glucoamylase complexed with P2, it was found that the residues Lys963, Tyr967, Asp969, Glu970, Ser991, Phe995, Trp1148, Lys1163, His1449, and Glu1451 were found to be involved in making hydrogen bonds with the peptide P2 and stabilized the complex. Similarly, the residues Pro994 and Trp1148 were found to be involved in hydrophobic interactions with P2 (Fig 13b). in addition to these interactions, a great number of residues were involved in water bridges in both complexes. The protein-peptide contact histograms of JNK1 in complex with P6 and pancreatic alpha-amylase in complex with P6 are shown in S2 Fig.

**Fig 14:**
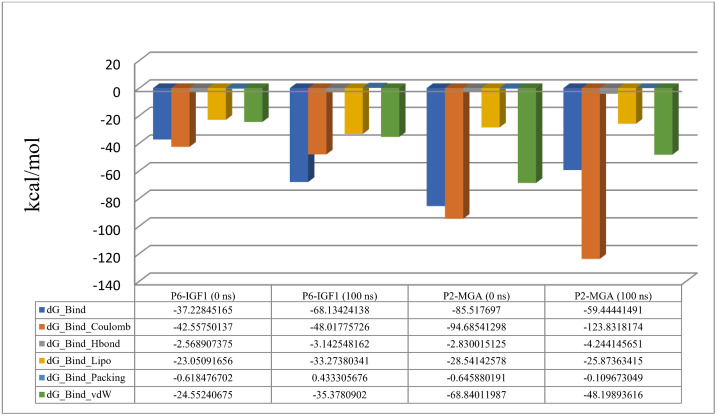
Molecular Mechanics Generalized Born Surface Area (MM-GBSA) calculated before and after the simulation. IGF1: Insulin-like growth factor 1 receptor; MGA: maltase-glucoamylase.

The Molecular Mechanics Generalized Born Surface Area (MM-GBSA) was also calculated for MD simulation using module of Primer MMGBSA v3.000 (Fig 14). To analyze MM-GBSA, solvation model VSGB and force field OPLS_2005 were used. For P6 and insulin-like growth factor 1 receptor complex, the average ΔG was found to be −31.07, the ΔG range was −37.22 to −68.13, and the standard deviation was determined to 47.96. Similarly, for P2 and maltase-glucoamylase complex, the average ΔG was calculated to be −38.23 kcal/mol with ΔG range of −85.51 to −59.44 and the standard deviation of 35.32. Most of the residues of the selected receptor proteins near their binding sites with the peptides contributed significantly in the peptide stability, which is seen by lower binding energy values.

In the current study, molecular dynamics simulation was used to validate the stability of the protein-peptide complexes with respect to the binding sites of the receptor proteins. The MD simulation method serves as a validation method to check the stability of a protein complexed with ligands [72]. The MD simulation can be used in a controlled environment (e.g., the human body) for the evaluation and the stability of a protein-ligand complex [73]. On the basis of findings of peptide screening, molecular docking was conducted to reveal interactions of top screened peptides against different selected receptor proteins. Top peptides (i.e., P2 and P6) with strong binding scores were further selected for molecular dynamics simulation study to exhibit the binding stability of the protein-peptide complexes.

Many biologically active substances such as proteins, peptides, and secondary metabolites have been found to be abundant in medicinal plants. The bulk of these substances which are used as medications are classified as secondary metabolites and their derivatives. Certain unavoidable limitations such as low abundance, toxicity, complexed multi-step synthesis, production specific to developmental stages, poor bioavailability, poor absorption, improper metabolism, and related adverse health issues have been linked to these secondary metabolite-based medications [74]. The scientific community is now compelled to look for other physiologically active molecules like proteins and peptides in plants due to the inevitable limitations that are associated to secondary metabolites. Peptides derived from medicinal plants are noted to be potential therapeutically active candidates [74]. Owing to these inevitable constraints, the production of bioactive peptides from proteins derived from plants has drawn increased interest because of their physiological and functional properties, which are crucial for both improving human health and reducing chronic illnesses. In addition to offering substantial nutrients, they have number of peptide sequences that have particular physiological advantages [75].

## Conclusion

The docking analysis of selected peptides have revealed their potential as drug candidates against receptor proteins to control diabetes. In the current study, haddock 2.4 was employed for protein-peptide docking analysis. The peptides P2 (CYYNNYSMSL) and P6 (LGPNNGYYYGGHANGSSIGM) were found to be the peptides which showed strong interactions with the selected receptor proteins, having haddock scores of −108.7 + /- 9.3 kcal/mol against maltase-glucoamylase and −121.3 + /- 3.1 kcal/mol against pancreatic alpha-amylase receptors, respectively. The P2 showed strong binding interactions with haddock score of The MD simulation of 100 ns also exhibited that the peptides P2 and P6 were remained firmly bound to proteins maltase-glucoamylase and insulin-like growth factor 1 receptor (haddock score of −101.5 + /- 5.4 kcal/mol), respectively for the simulation time. The peptides P2 and P6, validated through molecular docking and MD simulations, revealed promise as multi-target antidiabetic drug candidates but require in vitro/in vivo validation and formulation optimization, such as nanoparticle delivery to enhance their stability and bioavailability for clinical translation. The study may serve as a first step towards the future applications of these peptides as potential medications to treat diabetes mellitus.

## Supporting information

S1 Table List of peptides generated using Peptide Cutter Server digested by trypsin and pepsin enzymes. (PDF)

S2 Table Characteristics of peptides predicted by Peptide Ranker and Toxin-Pred tools. (PDF)

S1 Fig MD simulation showing RMSD trajectories of P6 with JNK1 and α-amylase. The MD simulation of the docked complex of (a) P6- c-Jun N-terminal kinase 1 (JNK1) and (b) P6-pancreatic alpha-amylase.
(TIF)

S2 Fig Protein-peptide contact histograms. (a) JNK1 complexed with P6; (b) Pancreatic alpha-amylase complexed with P6.
(TIF)

## References

[1] Arif R, Ahmad S, Mustafa G, Mahrosh HS, Ali M, Tahir Ul Qamar M, et al. Molecular docking and simulation studies of antidiabetic agents devised from hypoglycemic polypeptide-P of momordica charantia. Biomed Res Int. 2021;2021:5561129. doi: 10.1155/2021/5561129 34589547 PMC8476269

[2] Votey SR, Peters AL. Diabetes mellitus, type 2 - A review. 2005 [Accessed 2006 December 5].

[3] Piero M, Nzaro G, Njagi J. Diabetes mellitus-a devastating metabolic disorder. 2015.

[4] Baynes H. Classification, pathophysiology, diagnosis and management of diabetes mellitus. J diabetes metab. 2015;6:1–9.

[5] Patil SP, Goswami A, Kalia K, Kate AS. Plant-derived bioactive peptides: a treatment to cure diabetes. Int J Pept Res Ther. 2020;26(2):955–68. doi: 10.1007/s10989-019-09899-z 32435169 PMC7223764

[6] Tolman KG, Chandramouli J. Hepatotoxicity of the thiazolidinediones. Clin Liver Dis. 2003;7(2):369–79, vi. doi: 10.1016/s1089-3261(03)00020-5 12879989

[7] Yuan X, Gu X, Tang J. Purification and characterisation of a hypoglycemic peptide from Momordica Charantia L. Var. abbreviata Ser. Food Chem. 2008;111(2):415–20. doi: 10.1016/j.foodchem.2008.04.006 26047444

[8] Krisanapun C, Lee S-H, Peungvicha P, Temsiririrkkul R, Baek SJ. Antidiabetic Activities of Abutilon indicum (L.) sweet are mediated by enhancement of adipocyte differentiation and activation of the GLUT1 promoter. Evid Based Complement Alternat Med. 2011;2011:167684. doi: 10.1093/ecam/neq004 21603234 PMC3094712

[9] Pandey A, Tripathi P, Pandey R, Srivatava R, Goswami S. Alternative therapies useful in the management of diabetes: a systematic review. J Pharm Bioallied Sci. 2011;3(4):504–12. doi: 10.4103/0975-7406.90103 22219583 PMC3249697

[10] Hartmann R, Meisel H. Food-derived peptides with biological activity: from research to food applications. Curr Opin Biotechnol. 2007;18(2):163–9. doi: 10.1016/j.copbio.2007.01.013 17292602

[11] Sajal H, Patil SM, Raj R, Shbeer AM, Ageel M, Ramu R. Computer-aided screening of phytoconstituents from ocimum tenuiflorum against diabetes mellitus targeting DPP4 inhibition: a combination of molecular docking, molecular dynamics, and pharmacokinetics approaches. Molecules. 2022;27(16):5133. doi: 10.3390/molecules27165133 36014373 PMC9415412

[12] Korhonen H, Pihlanto A. Bioactive peptides: production and functionality. Int Dairy J. 2006;16(9):945–60. doi: 10.1016/j.idairyj.2005.10.012

[13] Agyei D, Ongkudon CM, Wei CY, Chan AS, Danquah MK. Bioprocess challenges to the isolation and purification of bioactive peptides. Food Bioproducts Process. 2016;98:244–56. doi: 10.1016/j.fbp.2016.02.003

[14] Roy S, Teron R, Nikku Linga R. PhytoSelectDBT: a database for the molecular models of anti-diabetic targets docked with bioactive peptides from selected ethno-medicinal plants. Bioinformation. 2023;19(9):908–17. doi: 10.6026/97320630019908 37928486 PMC10625370

[15] Saldívar-González FI, Navarrete-Vázquez G, Medina-Franco JL. Design of a multi-target focused library for antidiabetic targets using a comprehensive set of chemical transformation rules. Front Pharmacol. 2023;14:1276444. doi: 10.3389/fphar.2023.1276444 38027021 PMC10651762

[16] Rao MMV, Hariprasad TPN. In silico analysis of a potential antidiabetic phytochemical erythrin against therapeutic targets of diabetes. In Silico Pharmacol. 2021;9(1):5. doi: 10.1007/s40203-020-00065-8 33442532 PMC7779391

[17] Taghipour MJ, Ezzatpanah H, Ghahderijani M. In vitro and in silico studies for the identification of anti-cancer and antibacterial peptides from camel milk protein hydrolysates. PLoS One. 2023;18(7):e0288260. doi: 10.1371/journal.pone.0288260 37437001 PMC10337890

[18] Gupta S, Kapoor P, Chaudhary K, Gautam A, Kumar R, Open Source Drug Discovery Consortium, et al. In silico approach for predicting toxicity of peptides and proteins. PLoS One. 2013;8(9):e73957. doi: 10.1371/journal.pone.0073957 24058508 PMC3772798

[19] Shen Y, Maupetit J, Derreumaux P, Tufféry P. Improved PEP-FOLD approach for peptide and miniprotein structure prediction. J Chem Theory Comput. 2014;10(10):4745–58. doi: 10.1021/ct500592m 26588162

[20] Mahrosh HS, Mehmood R, Bukhari SA, Afzal G, Arif R. Investigation of hypoglycemic peptides derived from conserved regions of adMc1 to reveal their antidiabetic activities. Biomed Res Int. 2021;2021:5550180. doi: 10.1155/2021/5550180 33763471 PMC7963905

[21] Honorato RV, Trellet ME, Jiménez-García B, Schaarschmidt JJ, Giulini M. The HADDOCK2.4 web server for integrative modeling of biomolecular complexes. Nature Protocols. 2024;19:3219–41. 38886530 10.1038/s41596-024-01011-0

[22] DeLano WL. Pymol: an open-source molecular graphics tool. CCP4 Newsl Protein Crystallogr. 2002;40:82–92.

[23] Laskowski RA. PDBsum new things. Nucleic Acids Res. 2009;37(Database issue):D355–9. doi: 10.1093/nar/gkn860 18996896 PMC2686501

[24] Mustafa G, Mehmood R, Mahrosh HS, Mehmood K, Ahmed S. Investigation of plant antimicrobial peptides against selected pathogenic bacterial species using a peptide-protein docking approach. Biomed Res Int. 2022;2022:1077814. doi: 10.1155/2022/1077814 35355819 PMC8960006

[25] Shivakumar D, Williams J, Wu Y, Damm W, Shelley J, Sherman W. Prediction of absolute solvation free energies using molecular dynamics free energy perturbation and the OPLS force field. J Chem Theory Comput. 2010;6(5):1509–19. doi: 10.1021/ct900587b 26615687

[26] Mustafa G, Mahrosh HS, Salman M, Ali M, Arif R. In silico analysis of honey bee peptides as potential inhibitors of Capripoxvirus DNA-directed RNA polymerase. Animals. 2023;13:2281. 37508058 10.3390/ani13142281 PMC10376589

[27] Sedov IA, Zuev YF. Recent advances in protein-protein interactions. Int J Mol Sci. 2023;24(2):1282. doi: 10.3390/ijms24021282 36674795 PMC9864157

[28] Rao VS, Srinivas K, Sujini GN, Kumar GNS. Protein-protein interaction detection: methods and analysis. Int J Proteomics. 2014;2014:147648. doi: 10.1155/2014/147648 24693427 PMC3947875

[29] van Dijk M, van Dijk ADJ, Hsu V, Boelens R, Bonvin AMJJ. Information-driven protein-DNA docking using HADDOCK: it is a matter of flexibility. Nucleic Acids Res. 2006;34(11):3317–25. doi: 10.1093/nar/gkl412 16820531 PMC1500871

[30] Spiliotopoulos D, Kastritis PL, Melquiond ASJ, Bonvin AMJJ, Musco G, Rocchia W, et al. dMM-PBSA: a new HADDOCK scoring function for protein-peptide docking. Front Mol Biosci. 2016;3:46. doi: 10.3389/fmolb.2016.00046 27630991 PMC5006095

[31] Agarwal P, Gupta R. Alpha-amylase inhibition can treat diabetes mellitus. Res Rev J Med Health Sci. 2016;5:1–8.

[32] Kaur N, Kumar V, Nayak SK, Wadhwa P, Kaur P, Sahu SK. Alpha-amylase as molecular target for treatment of diabetes mellitus: a comprehensive review. Chem Biol Drug Des. 2021;98(4):539–60. doi: 10.1111/cbdd.13909 34173346

[33] Ponnusamy S, Haldar S, Mulani F, Zinjarde S, Thulasiram H, RaviKumar A. Gedunin and azadiradione: human pancreatic alpha-amylase inhibiting limonoids from neem (Azadirachta indica) as anti-diabetic agents. PLoS One. 2015;10(10):e0140113. doi: 10.1371/journal.pone.0140113 26469405 PMC4607367

[34] Settu R, Selvaraj D, Padikasan IA. GCMS profiling and in silico screening of alpha-amylase inhibitors in traditional pigmented rice varieties (Oryza sativa Linn) of Tamil Nadu. Food Bioscience. 2021;42:101154. doi: 10.1016/j.fbio.2021.101154

[35] Xavier TF, Sabitha R, Balavivekananthan S. In vitro pharmacological investigations of Oxystelma esculentum R.Br. and in silico molecular docking analysis of its leaf constituents on diabetic related target. South African J Botany. 2022;149:320–38. doi: 10.1016/j.sajb.2022.06.021

[36] Biadgo B, Tamir W, Ambachew S. Insulin-like growth factor and its therapeutic potential for diabetes complications - mechanisms and metabolic links: a review. Rev Diabet Stud. 2020;16(1):24–34. doi: 10.1900/RDS.2020.16.24 33905470 PMC9380093

[37] LeRoith D, Yakar S. Mechanisms of disease: metabolic effects of growth hormone and insulin-like growth factor 1. Nat Clin Pract Endocrinol Metab. 2007;3(3):302–10. doi: 10.1038/ncpendmet0427 17315038

[38] Singh RK, Chaurasiya AK, Kumar A. Ab initio modeling of human IRS1 protein to find novel target to dock with drug MH to mitigate T2DM diabetes by insulin signaling. 3 Biotech. 2024;14:108. 10.1007/s13205-024-03955-2 PMC10925585 38476643

[39] Damián-Medina K, Salinas-Moreno Y, Milenkovic D, Figueroa-Yáñez L, Marino-Marmolejo E, Higuera-Ciapara I, et al. In silico analysis of antidiabetic potential of phenolic compounds from blue corn (Zea mays L.) and black bean (Phaseolus vulgaris L.). Heliyon. 2020;6(3):e03632. doi: 10.1016/j.heliyon.2020.e03632 32258479 PMC7110303

[40] Thakur S, Gupta SK, Ali V, Singh P, Verma M. Aldose Reductase: a cause and a potential target for the treatment of diabetic complications. Arch Pharm Res. 2021;44(7):655–67. doi: 10.1007/s12272-021-01343-5 34279787

[41] James JP, Fabin AM, Sasidharan P, Kumar P. Virtual screening, molecular docking and pharmacophore modeling of phytoconstituents of flavones as aldose reductase inhibitors. JPRI. 2021:94–107. doi: 10.9734/jpri/2021/v33i41b32348

[42] Halayal RY, Bagewadi ZK, Maliger RB, Al Jadidi S, Deshpande SH. Network pharmacology based anti-diabetic attributes of bioactive compounds from Ocimum gratissimum L. through computational approach. Saudi J Biol Sci. 2023;30(9):103766. doi: 10.1016/j.sjbs.2023.103766 37588570 PMC10425415

[43] Mulvihill EE, Drucker DJ. Pharmacology, physiology, and mechanisms of action of dipeptidyl peptidase-4 inhibitors. Endocr Rev. 2014;35(6):992–1019. doi: 10.1210/er.2014-1035 25216328 PMC7108477

[44] Sivaraman SA, Sabareesh V. An update on dipeptidyl peptidase-IV inhibiting peptides. Curr Protein Pept Sci. 2024;25(4):267–85. doi: 10.2174/0113892037287976231212104607 38173201

[45] Nongonierma AB, Mooney C, Shields DC, FitzGerald RJ. In silico approaches to predict the potential of milk protein-derived peptides as dipeptidyl peptidase IV (DPP-IV) inhibitors. Peptides. 2014;57:43–51. doi: 10.1016/j.peptides.2014.04.018 24793774

[46] Cian RE, Nardo AE, Garzón AG, Añon MC, Drago SR. Identification and in silico study of a novel dipeptidyl peptidase IV inhibitory peptide derived from green seaweed Ulva spp. hydrolysates. LWT. 2022;154:112738. doi: 10.1016/j.lwt.2021.112738

[47] Khan A, Unnisa A, Sohel M, Date M, Panpaliya N, Saboo SG, et al. Investigation of phytoconstituents of Enicostemma littorale as potential glucokinase activators through molecular docking for the treatment of type 2 diabetes mellitus. In Silico Pharmacol. 2021;10(1):1. doi: 10.1007/s40203-021-00116-8 34926125 PMC8639997

[48] Thilagavathi R, Hosseini-Zare MS, Malini M, Selvam C. A comprehensive review on glucokinase activators: Promising agents for the treatment of Type 2 diabetes. Chem Biol Drug Des. 2022;99(2):247–63. doi: 10.1111/cbdd.13979 34714587

[49] Matschinsky FM. Assessing the potential of glucokinase activators in diabetes therapy. Nat Rev Drug Discov. 2009;8(5):399–416. doi: 10.1038/nrd2850 19373249

[50] Rathinavel T, Ammashi S, Gnanendra Shanmugam ST. Identification of anti-diabetic phytocompounds from Ficus racemosa and its validation through in silico molecular modeling. Int J Adv Sci Eng. 2019;5:1085–98.

[51] Bennett BL, Satoh Y, Lewis AJ. JNK: a new therapeutic target for diabetes. Curr Opin Pharmacol. 2003;3(4):420–5. doi: 10.1016/s1471-4892(03)00068-7 12901952

[52] Sabio G, Davis RJ. cJun NH2-terminal kinase 1 (JNK1): roles in metabolic regulation of insulin resistance. Trends Biochem Sci. 2010;35(9):490–6. doi: 10.1016/j.tibs.2010.04.004 20452774 PMC2975251

[53] Kaneto H, Matsuoka T, Nakatani Y, Kawamori D, Matsuhisa M, Yamasaki Y. Oxidative stress and the JNK pathway in diabetes. Curr Diabetes Rev. 2005;1(1):65–72. doi: 10.2174/1573399052952613 18220583

[54] Mandar BK, Khanal P, Patil BM, Dey YN, Pasha I. In silico analysis of phytoconstituents from Tinospora cordifolia with targets related to diabetes and obesity. In Silico Pharmacol. 2021;9(1):3. doi: 10.1007/s40203-020-00063-w 33442530 PMC7778662

[55] Kurniawan R, Taslim NA, Hardinsyah H, Syauki AY, Idris I, Aman AM, et al. Pharmacoinformatics and cellular studies of algal peptides as functional molecules to modulate type-2 diabetes markers. Future Foods. 2024;9:100354. doi: 10.1016/j.fufo.2024.100354

[56] Ren L, Cao X, Geng P, Bai F, Bai G. Study of the inhibition of two human maltase-glucoamylases catalytic domains by different α-glucosidase inhibitors. Carbohydr Res. 2011;346(17):2688–92. doi: 10.1016/j.carres.2011.09.012 22036121

[57] Sumaryada T, Roslia A, Ambarsari L, Kartono A. Molecular docking simulation of mangostin derivatives and curcuminoid on maltase-glucoamylase target for searching anti-diabetes drug candidates. IEEE; 2016: 1–4.

[58] Guan S, Han X, Li Z, Xu X, Cui Y, Chen Z, et al. Exploration of the Interactions between Maltase–Glucoamylase and its potential peptide inhibitors by molecular dynamics simulation. Catalysts. 2022;12(5):522. doi: 10.3390/catal12050522

[59] Abdulhaniff P, Sakayanathan P, Loganathan C, Iruthayaraj A, Thiyagarajan R, Thayumanavan P. Mammalian maltase-glucoamylase and sucrase-isomaltase inhibitory effects of Artocarpus heterophyllus: An in vitro and in silico approach. Comput Biol Chem. 2024;110:108052. doi: 10.1016/j.compbiolchem.2024.108052 38492557

[60] Rangwala SM, Lazar MA. Peroxisome proliferator-activated receptor gamma in diabetes and metabolism. Trends Pharmacol Sci. 2004;25(6):331–6. doi: 10.1016/j.tips.2004.03.012 15165749

[61] Bermúdez V, Finol F, Parra N, Parra M, Pérez A, Peñaranda L, et al. PPAR-gamma agonists and their role in type 2 diabetes mellitus management. Am J Ther. 2010;17(3):274–83. doi: 10.1097/MJT.0b013e3181c08081 20216208

[62] Yadav M, Yadav P, Yadav JP, Kataria SK. Novel derivatives of Costus igneus towards potentiality against diabetes mellitus receptors: ADME/Tox profiling, computational docking, and molecular dynamics simulation study. J Taibah Univ Sci. 2024;18(1). doi: 10.1080/16583655.2024.2370107

[63] Li S, Shin HJ, Ding EL, van Dam RM. Adiponectin levels and risk of type 2 diabetes: a systematic review and meta-analysis. JAMA. 2009;302(2):179–88. doi: 10.1001/jama.2009.976 19584347

[64] Spranger J, Kroke A, Möhlig M, Bergmann MM, Ristow M, Boeing H, et al. Adiponectin and protection against type 2 diabetes mellitus. Lancet. 2003;361(9353):226–8. doi: 10.1016/S0140-6736(03)12255-6 12547549

[65] Khanal P, Mandar BK, Patil BM, Hullatti KK. In silico antidiabetic screening of borapetoside C, cordifolioside A and magnoflorine. Pharm Sci. 2019;81(3). doi: 10.36468/pharmaceutical-sciences.543

[66] Zhao F-Q, Keating AF. Functional properties and genomics of glucose transporters. Curr Genomics. 2007;8(2):113–28. doi: 10.2174/138920207780368187 18660845 PMC2435356

[67] Pragallapati S, Manyam R. Glucose transporter 1 in health and disease. J Oral Maxillofac Pathol. 2019;23(3):443–9. doi: 10.4103/jomfp.JOMFP_22_18 31942129 PMC6948067

[68] Lu L, Seidel CP, Iwase T, Stevens RK, Gong Y-Y, Wang X, et al. Suppression of GLUT1; a new strategy to prevent diabetic complications. J Cell Physiol. 2013;228(2):251–7. doi: 10.1002/jcp.24133 22717959

[69] Chaudhary RK, Karoli SS, Dwivedi PSR, Bhandari R. Anti-diabetic potential of Corn silk (Stigma maydis): An in-silico approach. J Diabetes Metab Disord. 2022;21(1):445–54. doi: 10.1007/s40200-022-00992-7 35673494 PMC9167408

[70] Rathore PK, Arathy V, Attimarad VS, Kumar P, Roy S. In-silico analysis of gymnemagenin from Gymnema sylvestre (Retz.) R.Br. with targets related to diabetes. J Theor Biol. 2016;391:95–101. doi: 10.1016/j.jtbi.2015.12.004 26711684

[71] Rasheed MA, Iqbal MN, Saddick S, Ali I, Khan FS, Kanwal S, et al. Identification of Lead Compounds against Scm (fms10) in Enterococcus faecium Using Computer Aided Drug Designing. Life (Basel). 2021;11(2):77. doi: 10.3390/life11020077 33494233 PMC7909823

[72] Aljahdali MO, Molla MHR, Ahammad F. Compounds identified from marine mangrove plant (Avicennia alba) as potential antiviral drug candidates against WDSV, an In-Silico approach. Mar Drugs. 2021;19(5):253. doi: 10.3390/md19050253 33925208 PMC8145693

[73] Shah A, Ahmad I, Ahmad I, Amin A, Rather MA. Gene characterization, molecular docking and dynamic simulations of insulin-like growth factor receptor (IGF-1Ra) in common carp, Cyprinus carpio. Springer; 2023: 382–96.

[74] Wani SS, Dar PA, Zargar SM, Dar TA. Therapeutic potential of medicinal plant proteins: present status and future perspectives. Curr Protein Pept Sci. 2020;21(5):443–87. doi: 10.2174/1389203720666191119095624 31746291

[75] Ajayi FF, Mudgil P, Jobe A, Antony P, Vijayan R, Gan C-Y, et al. Novel Plant-Protein (Quinoa) derived bioactive peptides with potential anti-hypercholesterolemic activities: identification, characterization and molecular docking of bioactive peptides. Foods. 2023;12(6):1327. doi: 10.3390/foods12061327 36981252 PMC10048307

