# Supplementary material for: Characterization of multi-targeted insulin-mimetic antidiabetic peptides using in silico approaches
Source: PLoS One. 2025 Aug 19;20(8):e0330341. doi: 10.1371/journal.pone.0330341 (PMC12364323; doi:10.1371/journal.pone.0330341)
Supplement: S1 Table — (PDF) [file pone.0330341.s001.pdf]

**S1 Table. List of peptides generated using Peptide Cutter Server digested by trypsin and pepsin enzymes.**

| Sr. No | Sequence |
|---|---|
| | Cq-IMP Cnidoscolus quercifolius |
| 1. | TK |
| 2. | DPEL |
| 3. | QCK |
| 4. | QQK |
| 5. | 1 QQQYDDDDK |
| | AdMc1 protein |
| 6. | 2 SSIIA |
| 7. | AVA |
| 8. | 3 IADAYAYR |
| 9. | 4 TTITTVEVDEDNQGR |
| 10. | QER |
| 11. | 5 HIRPR |
| 12. | EQL |
| 13. | SCQD |
| 14. | 6 QQGGGR |
| 15. | EIL |
| 16. | 7 ENQWGR |
| 17. | EQG |
| 18. | L |
| 19. | 8 EECCR |
| 20. | 9 NVEEQCR |
| 21. | CDA |
| 22. | L |
| 23. | 10 EEVAR |
| 24. | 11 EVQSQQHGQQGSQI |
| 25. | L |
| 26. | QHAR |
| 27. | 12 MLPSMCQIRPQR |
| 28. | CDF |
| | polypeptide-P |
| 29. | 13 MGCDEA |
| 30. | 14 STPGVIPTR |
| 31. | 15 HMDDPTGGVC |
| 32. | 16 YESIR |
| 33. | 17 DTSEPGAVA |
| 34. | DEGK |
| 35. | 18 VDESMTDGK |
| 36. | DDGH |
| 37. | 19 KPVAGD |
| 38. | VAEK |

| | |
|---|---|
| 39. | 20 FGASGDVECITP |
| 40. | 21 FGDTDE |
| 41. | 22 LEPGGGG |
| 42. | 23 IVAPSTDR |
| 43. | LK |
| 44. | SPLF |
| 45. | 24 VAESAE |
| 46. | 25 HASEVK |
| 47. | 26 SIHEPEA |
| 48. | 27 ADSEGR |
| 49. | 28 FLAGEK |
| | Prolamin binding factor |
| 50. | MEEV |
| 51. | 29 SSNSK |
| 52. | 30 AGQMAGEAAAAAEK |
| 53. | K |
| 54. | 31 SRPKPEQK |
| 55. | 32 VECPR |
| 56. | 32 SGNTK |
| 57. | 33 CYYNNYSMSL |
| 58. | 34 YWTHGGS |
| 59. | 35 NVPIGGGCRKPK |
| 60. | 36 RPGTSDAHK |
| 61. | 37 LGMASSSEPTGVVPPSNCTGMN |
| 62. | ANVL |
| 63. | 38 PTFMSGG |
| 64. | 39 DIQSS |
| 65. | 40 GSSSSSNPTA |
| 66. | L |
| 67. | 41 MSPGGTTS |
| 68. | 42 GGAGG |
| 69. | DGS |
| 70. | 43 LGPNNGYYYGGHANGSSIGM |
| 71. | 44 MTPPTVS |
| 72. | 45 GIPSPMQQHGGL |
| 73. | 46 VVGGNGIGGTTSST |
| 74. | 47 QGSAGEEGDDGTGSIMG |
| 75. | 48 QWQPHVGNGGGGVVG |
| 76. | 49 GGAHH |
| 77. | 50 LGTGNNVTMGNNNNNNNQNNNNGGGAGDDDDGGSSR |
| 78. | 51 DCYWINNGGSNPWQS |
| 79. | NSTS |
| | MC6 |
| 80. | TNMK |
| 81. | 52 HMAGAAAAGAVVG |

| | |
|---|---|
| | Charantin |
| 82. | 53 NVEQQCR |
| 83. | 54 IHGQQ |
| | Trypsin inhibitor BGIT |
| 84. | SR |
| 85. | CQGK |
| 86. | SSWPQL |
| 87. | VGSTGAAAK |
| 88. | 55 AVIER |
| 89. | ENPR |
| 90. | VR |
| 91. | AVIIK |
| 92. | VGSGATK |
| 93. | D |
| 94. | FR |
| 95. | CDR |
| 96. | VR |
| 97. | VWVTER |
| 98. | GIVARPPTIG |
| | MC2-1-5 |
| 99. | KTNMKHMAGAAAAGAVVG |
| | M.Cy protein |
| 100. | GLEPTTT |
