# Supplementary material for: Characterization of multi-targeted insulin-mimetic antidiabetic peptides using in silico approaches
Source: PLoS One. 2025 Aug 19;20(8):e0330341. doi: 10.1371/journal.pone.0330341 (PMC12364323; doi:10.1371/journal.pone.0330341)
Supplement: S2 Table — (PDF) [file pone.0330341.s002.pdf]

S2 Table. Characteristics of peptides predicted by Peptide Ranker and Toxin-Pred tools.

Peptides sequence	PeptideRanker score	Svm score	Prediction	Hydrophobicity	Side bulk	Charge	PI	MW
P1 SSWPQL	0.833612	-1.00	Non-Toxin	-0.06	0.00	0.00	5.24	716.79
P2 CYYNNYSMSL	0.602973	-0.49	Non-Toxin	-0.09	0.66	0.00	5.84	1257.53
P3 NVPIGGGCRKPK	0.512157	-0.57	Non-Toxin	-0.25	0.63	3.00	10.07	1225.65
P4 PTFMSGG	0.621438	-0.86	Non-Toxin	0.10	0.61	0.00	5.88	695.88
P5 GGAGG	0.52423	-0.81	Non-Toxin	0.18	0.65	0.00	5.88	317.37
P6 LGPNNGYYYGGHANGSSIGM	0.637122	-0.39	Non-Toxin	-0.01	0.62	0.50	7.08	2029.48
P7 GIPSPMQQHGGL	0.808791	-1.27	Non-Toxin	-0.01	0.55	0.50	7.10	1221.59
P8 QWQPHVGNGGGGVVG	0.525456	-1.15	Non-Toxin	0.03	0.61	0.50	7.10	1448.81
P9 QQQYDDDDK	0.169661	-0.79	Non-Toxin	-0.67	0.72	-3.00	3.77	1154.23
P10 SSIIA	0.225589	-0.77	Non-Toxin	0.24	0.60	0.00	5.88	489.63
P11 IADAYAYR	0.412616	-0.75	Non-Toxin	-0.12	0.64	0.00	6.18	942.13
P12 TTITTVEVDEDNQGR	0.0239639	-0.86	Non-Toxin	-0.30	0.66	-3.00	3.92	1677.96
P13 HIRPR	0.492621	-0.86	Non-Toxin	-0.65	0.48	2.50	12.01	677.87
P14 QQGGGR	0.434962	-0.69	Non-Toxin	-0.44	0.68	1.00	10.11	601.71
P15 ENQWGR	0.481719	-0.81	Non-Toxin	-0.53	0.66	0.00	6.36	788.90
P16 EECCR	0.289777	0.31	Toxin	-0.58	0.66	-1.00	4.54	638.76
P17 NVEEQCR	0.0944135	-0.69	Non-Toxin	-0.54	0.69	-1.00	4.54	877.03
P18 EEVAR	0.0434746	-0.89	Non-Toxin	-0.44	0.65	-1.00	4.54	602.70
P19 EVQSQQHGQQGSQI	0.0676733	-1.32	Non-Toxin	-0.29	0.61	-0.50	5.25	1553.84
P20 MLPSMCQIRPQR	0.450039	-0.26	Non-Toxin	-0.29	0.61	2.00	10.38	1459.98
P21 MGCDEA	0.254671	-0.45	Non-Toxin	-0.10	0.67	-2.00	3.67	624.75
P22 STPGVIPTR	0.341845	-0.80	Non-Toxin	-0.12	0.56	1.00	10.11	927.19
P23 HMDDPTGGVC	0.497726	-0.96	Non-Toxin	-0.09	0.59	-1.50	4.20	1031.26
P24 YESIR	0.13804	-0.83	Non-Toxin	-0.38	0.66	0.00	6.35	666.79
P25 DTSEPGAVA	0.0922804	-0.94	Non-Toxin	-0.07	0.59	-2.00	3.67	845.97
P26 VDESMTDGK	0.155671	-0.72	Non-Toxin	-0.29	0.68	-2.00	4.03	981.16
P27 KPVAGD	0.155298	-0.91	Non-Toxin	-0.16	0.62	0.00	6.19	585.73
P28 FGASGDVECITP	0.446752	-0.28	Non-Toxin	0.05	0.62	-2.00	3.67	1195.47
P29 FGDTDE	0.193415	-0.69	Non-Toxin	-0.24	0.68	-3.00	3.50	682.71
P30 LEPGGGG	0.37148	-0.74	Non-Toxin	0.07	0.61	-1.00	4.00	585.72
P31 IVAPSTDR	0.142585	-1.09	Non-Toxin	-0.18	0.60	0.00	6.19	858.06
P32 VAESAE	0.0391652	-0.70	Non-Toxin	-0.08	0.60	-2.00	3.80	604.68
P33 HASEVK	0.0682633	-0.87	Non-Toxin	-0.27	0.52	0.50	7.10	669.81
P34 SIHEPEA	0.132327	-1.13	Non-Toxin	-0.14	0.50	-1.50	4.51	781.91
P35 ADSEGR	0.17921	-0.64	Non-Toxin	-0.49	0.64	-1.00	4.38	633.68

P36 FLAGEK	0.279076	-1.01	Non-Toxin	-0.03	0.63	0.00	6.35	663.85
P37 SSNSK	0.114607	-0.91	Non-Toxin	-0.50	0.61	1.00	9.11	521.58
P38 AGQMAGEAAAAAEK	0.168581	-1.29	Non-Toxin	-0.05	0.61	-1.00	4.54	1275.58
P39 SRPKPEQK	0.185977	-0.71	Non-Toxin	-0.71	0.58	2.00	10.01	969.21
P40 VECPR	0.287467	-0.68	Non-Toxin	-0.37	0.61	0.00	6.32	602.76
P41 YWTHGGS	0.345712	-0.82	Non-Toxin	-0.02	0.52	0.50	7.09	806.93
P42 RPGTSDAHK	0.146703	-1.04	Non-Toxin	-0.45	0.53	1.50	9.10	968.15
P43 LGMASSSEPTGVVPPSNCTGMN	0.443988	-0.61	Non-Toxin	-0.03	0.60	-1.00	4.00	2136.71
P44 DIQSS	0.0770726	-0.80	Non-Toxin	-0.24	0.64	-1.00	3.80	548.61
P45 GSSSSSNPTA	0.247597	-0.58	Non-Toxin	-0.18	0.55	0.00	5.88	893.98
P46 MSPGGTTS	0.175019	-0.86	Non-Toxin	-0.05	0.58	0.00	5.88	736.90
P47 MTPPTVS	0.128993	-1.00	Non-Toxin	0.01	0.54	0.00	5.88	731.95
P48 VVGGNGIGGTTSST	0.107025	-0.87	Non-Toxin	0.06	0.64	0.00	5.88	1206.49
P49 QGSAGEEGDDGTGSIMG	0.46342	-0.77	Non-Toxin	-0.11	0.66	-4.00	3.44	1567.81
P50 GGAHH	0.358672	-0.84	Non-Toxin	-0.05	0.53	0.00	5.88	477.55
P51 DCYWINNGGSNPWQS	0.615231	0.46	Toxin	-0.14	0.63	-1.00	3.80	1741.04
P52 HMAGAAAAGAVVG	0.231538	-0.96	Non-Toxin	0.22	0.56	0.50	7.10	1082.42
P53 NVEQQCR	0.124498	-0.88	Non-Toxin	-0.55	0.69	0.00	6.32	876.05
P54 IHGQQ	0.148938	-0.99	Non-Toxin	-0.18	0.55	0.50	7.10	581.71
P55 AVIER	0.0772677	-0.89	Non-Toxin	-0.17	0.66	0.00	6.36	586.75
P56 GLEPTTT	0.114239	-0.92	Non-Toxin	-0.08	0.55	-1.00	4.00	717.87

PI: Isoelectric Point, MW: Molecular Weight
