# Supplementary figures and images for: Characterization of multi-targeted insulin-mimetic antidiabetic peptides using in silico approaches
Source: PLoS One. 2025 Aug 19;20(8):e0330341. doi: 10.1371/journal.pone.0330341 (PMC12364323; doi:10.1371/journal.pone.0330341)

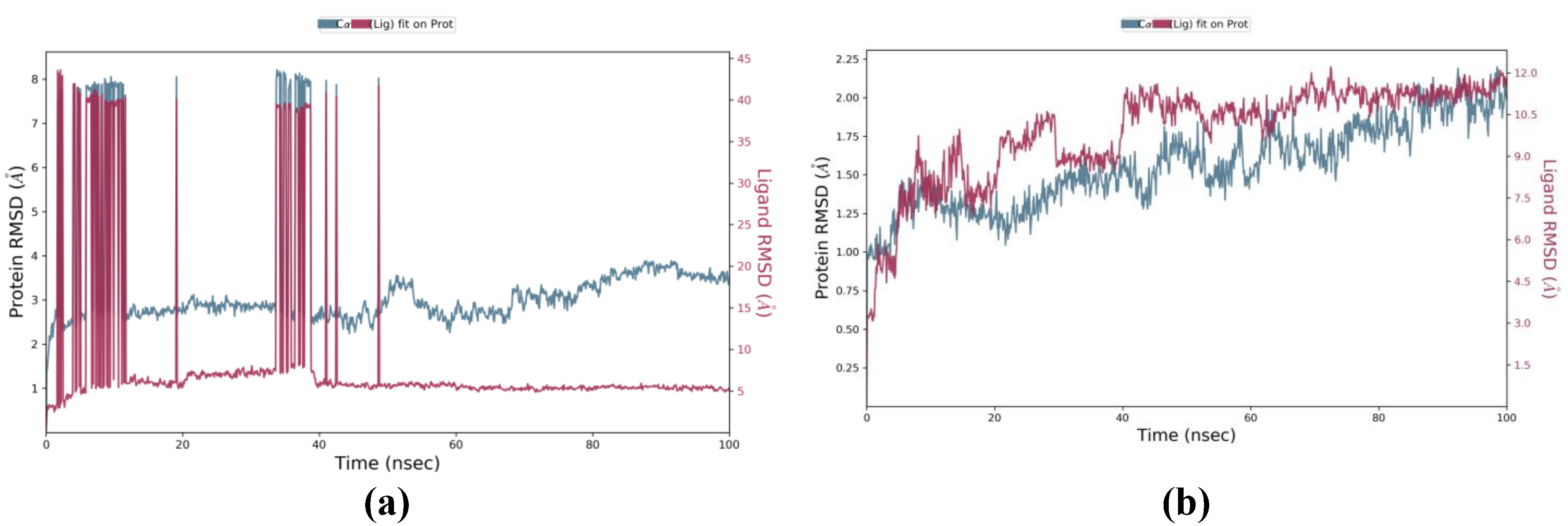

Supplement: S1 Fig — The MD simulation of the docked complex of (a) P6- c-Jun N-terminal kinase 1 (JNK1) and (b) P6-pancreatic alpha-amylase. (TIF) [file pone.0330341.s003.tif]

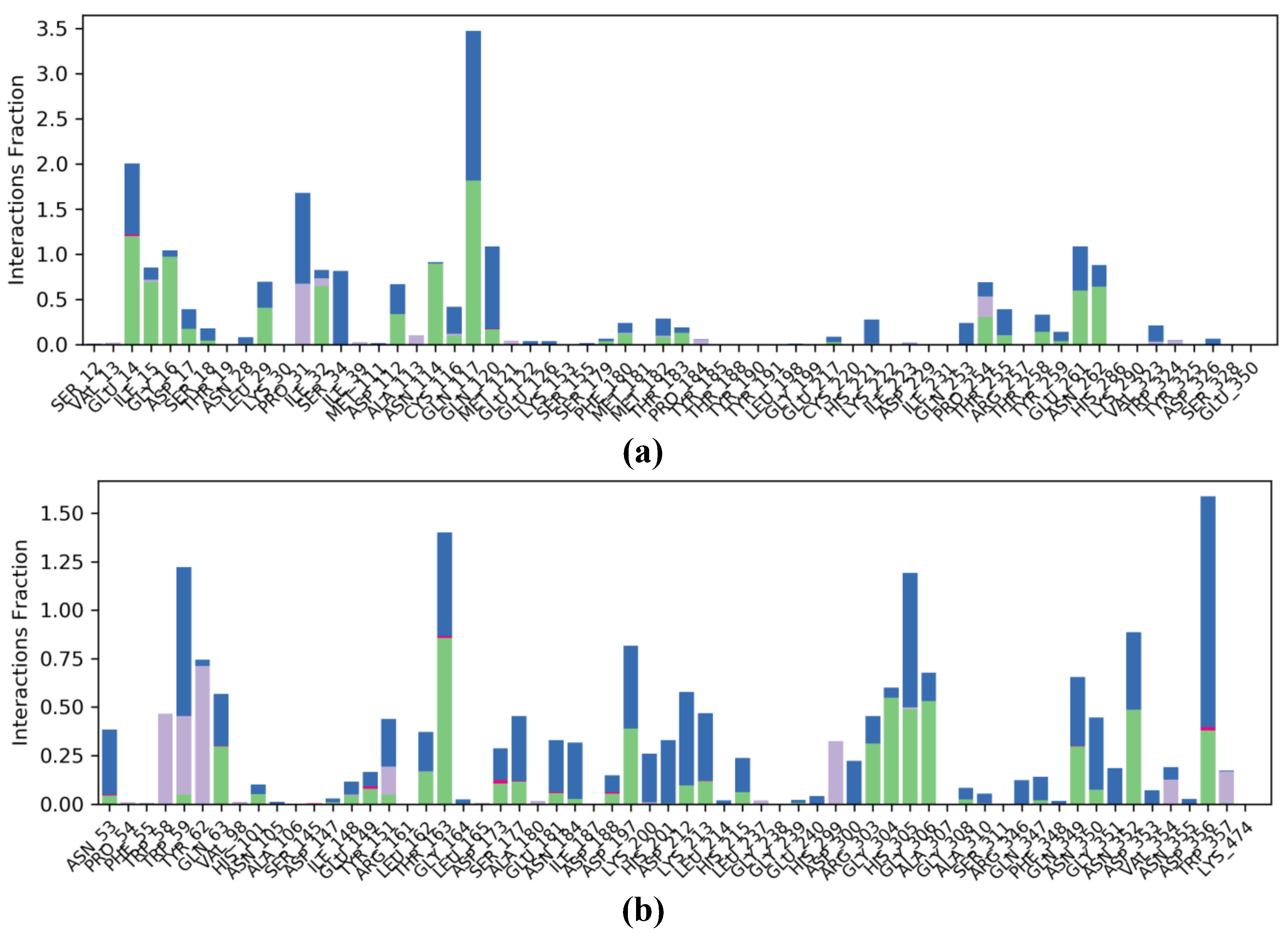

Supplement: S2 Fig — (a) JNK1 complexed with P6; (b) Pancreatic alpha-amylase complexed with P6. (TIF) [file pone.0330341.s004.tif]
